# Further Remarks on Venereal Complaints Different from Syphilis

**Published:** 1815-12

**Authors:** Richard Carmichael


*For the London Medical and Physical Journal.*

### *Further Remarks on Venereal Complaints different from Syphilis;* by Mr. Richard Carmichael.

THE form of venereal disease to which I mean, in this paper, to call your attention, is arranged in my Essay as a species of that class which produces the papular eruption. In my classification, I was chiefly influenced by the character of the constitutional eruptions, considering those different primary appearances which produce the same eruption as arising from the same poison. But at the time my work was committed to the press, I had not met with a single instance of a *primary ulcer without induration, but with elevated edges,* which was accompanied with its attendant eruption. I had, therefore, as I mentioned in my Essay, conjecture alone as my guide in classing the disorder in question among those which produce the papular eruption; and my subsequent experience has taught me that *that* conjecture was wrong.

This error was, however, rectified in the appendix to my work, in which I have stated, that, after my third chapter was printed, I had met with two instances of the primary ulcer with elevated edges, which were attended by a pustular eruption terminating in ulceration. The following is the passage: "The eruption occurred in the form of pustules in the neighbourhood of the primary ulcer, which, in a few days, extended, and became covered with flat crusts, and each spot extended to about the size of a sixpence. The crust quickly falling off, exposed a foul circular ulcer, exhibiting a regular concavity."

Since that period, three other cases have occurred of the same ulcer and its attendant constitutional eruption, which I shall detail in the sequel of this paper. In all of them the eruption was alike, and approached nearer to the character of of phlyzacia than any other cutaneous affection with which I am acquainted. According to Dr. Bateman, "a phlyzacium is a pustule of a large size, raised on a large circular base, of a vivid red colour, and succeeded by a thick, hard, dark-coloured scab." The scab, however, in those cases which came under my observation, was not thick, but in general of a thin scale-like appearance. In all other respects, each spot of the eruption coincided with the above definition of phlyzacium.

In the cases mentioned in my appendix, I omitted to note the character of the pustules; but, from the evidence of so limited a number of cases as I am about to detail, it might be too hasty to conclude, that the eruption attendant upon the primary ulcer with elevated edges is always formed of phlyzacia. As far, however, as my experience extends of this form of venereal disease, I have found that it is but seldom attended by any constitutional affection; and that when any eruption does occur, it consists in general of but a few phlyzacious pustules, some of which, after forming small thin scab-like crusts, gradually recede and disperse, while others enlarge, and form more extensive crusts, which at length falling off, expose ulcers that are of a circular form, and regularly excavated.

In one case only (XC) was there any affection of the throat; and in this instance it consisted of a general inflammation of the fauces, and a superficial ulcer on the left tonsil. In two or three instances, the patients complained of pains in their shoulders, arms, and thighs; but whether this poison is capable of producing nodes I cannot decide, not having met with any instance in which the ulcer with elevated edges was followed by that symptom.

A description of this ulcer, sufficiently minute to manifest its characters, is given in my Essay; and here I shall only observe, that its surface is destitute of any thing like granulations, and approaches in some degree the appearance of the phagedœnic ulcer, but is not by any means marked by the virulence and rapid progress of that most malignant of venereal complaints. The buboes arising from this poison have also high edges, and, when treated with mercury, often become most obstinate and inveterate ulcers, forming pendulous edges, which it is in vain to attempt to remove by caustic, or any means but the knife; but *this* I have often employed with advantage.

It may be here justly remarked, that the primary and secondary symptoms of a poison have a strong analogy to each other in their general character and progress. For instance—a true syphilitic chancre is indolent, and extremely slow slow in its progress, and the scaly eruption which follows is also equally indolent and slow in producing ulceration, often requiring weeks before that process ensues The ulcer with defined or elevated edges, the subject of this paper, is more active in its nature, and quicker in its progress, than chancre. The constitutional eruption is also more active than that of true syphilis, and proceeds to form crusts in the course of a few days after its appearance. The phagedenic and sloughing ulcers are the most active and rapid in their progress of venereal primary ulcers; and the pustules which form the constitutional ulcers are of so short an existence, that they in general escape the observation, not only of the medical attendant, but of the patient himself. The pustule, which first appears at night, may, in the morning, be an ulcer covered with a thick dark crust The constitutional ulcers also, like the primary, spread with quickness to a far greater extent than those of the other venereal poisons.

In a classification of venereal diseases, the character of the ulcer with elevated edges, as well as the pustular form of the eruption which it produces, would lead me to place it in a class by itself, there being no other known species of venereal disorder which produces similar constitutional symptoms. This CLASS would naturally *follow* that which comprehends the primary affections which excite a papular eruption; and *precede* that in which I have comprehended the phagedœnic and sloughing ulcers.

Before I proceed to the detail of my cases, I beg to remind the reader that the same abbreviations are made use of through this paper as were adopted in my last; that the cases are numbered, as in the same series; and that in the following selection he will only find

CASES *of* SUPERFICIAL ULCERS *without* INDURATION, *but with* ELEVATED EDGES.

Case LXXI.—Maurice Ivory, April 13th, 1813. An extensive ulcer engaging more than one half of the external surface of the prepuce; an ulcerated bubo in the right groin. He stated that he had been disordered seven weeks, and had not used mercury. Solut. Antim. Lot. Nig. Catapl. Inguini. May 10th, the ulcers, both of the penis and groin, were healed. Discharged the hospital.—On the 13th of the following December, he was re-admitted, on account of pains in his shoulders, arms, and thighs, for which he was directed to take a pill containing three grains of antimonial powder and half a grain of calomel, three times a-day. Dec. 25th, discharged well

Case LXXII.—Thomas Field, July 2d, 1813. Two ulcers, cers, the larger about the size of a sixpence, situated on the body of the penis, and the other on the glans; tenderness of the right groin. He stated that he had been a month disordered, and had not used mercury. Solut. Antim. Lot. flav. 26th, ulcers healed. Discharged the hospital well.

Case LXXIII.—Patrick Fitsimons, July 7th, 1813. An ulcer, somewhat larger than a shilling, situated on the urethral side of the body of the penis; a large bubo in the groin, containing matter. He stated that he had been eight weeks disordered, that a fortnight previous to his admission the bubo appeared, and that he had not used mercury. Solut. Antim. Lot. flav. Catapl. Inguini. 12th, the bubo had ulcerated; the ulcer on the penis was improving, and was completely healed before the 24th. The bubo at this time looked clear, but exhibited elevated edges, to which it became necessary to apply caustic daily. August 9th, the ulcer of the groin had also healed. Discharged the hospital well.

Case LXXIV.—Patrick Conolly, August 18, 1813. Two small ulcers situated on the dorsum penis, the larger about the size of a silver penny; a small bubo in the left groin. He stated that he had been disordered three weeks, and had used three drachms of mercurial ointment. Solut. Antim. Lot. flav. 27th, the ulcer of the penis healed; the bubo ulcerated, and now exhibited elevated edges, to which it became necessary to apply caustic repeatedly. Sept. 6th, the ulcer of the groin was healing. Discharged the hospital at his own request.

Case LXXV.—John M'Donald, August 25, 1813. An ulcer, the size of a shilling, on the dorsum penis, near the pubes; two smaller ulcers on the prepuce, with some fullness of that part; and a small fungous looking ulcer on the glans penis. He stated that he had been two months disordered previous to his admission, and had not used mercury. Solut. Antim. Lot. flav. Sept. 9th, the ulcers as before. The antimonial solution was discontinued, and a grain of calomel exhibited night and morning. 20th, his mouth was affected, but as yet no amendment in the state of the ulcers; the calomel was, therefore, laid aside, and he was directed to take five grains of extract of cicuta three times a-day. 27th, the ulcers still remaining stationary, recourse was again had to the antim. solut. October 4th, the ulcers had assumed a healing appearance, but a bubo had appeared in the left groin. 11th, the ulcer of the penis was healed; repeated blisters were applied to the bubo, which was hard and indolent. 25th, the bubo was nearly dispersed. Discharged the hospital.

Case LXXVI.—John O'Neil, Sept. 16, 1813. Two ulcers on the external surface of the prepuce, the larger about the extent and shape of a bean; his mouth was affected with mercury at the time of his admission, and he mentioned that the smaller ulcer appeared while he was under the influence of that medicine. Solut. Antim. Lot. flav. Oct. 11th, the ulcers were healed. Discharged the hospital.

Case LXXVII.—Patrick Hogg, March 4, 1814. Phymosis, inflammation and swelling of the penis, a circle of small ulcers round the orifice of the prepuce, discharge from beneath the prepuce. He stated that he had been three weeks disordered, and had not used mercury. Solut. Antim. Lot. flav. Fotus. Catapl. 14th, inflammation and swelling reduced, ulcers nearly healed, discharge under the prepuce had ceased. 21st, discharged the hospital well.

Case LXXVIII.—Thomas Gorman, April 8, 1814. A large ulcer on the external surface of the prepuce; a deep ulcerated bubo, with elevated edges, in each groin. He stated that he had been a month disordered, and had not used mercury. Solut. Antim. Lot. flav. The edges of the buboes touched daily with caustic. May 2d, the ulcer on the prepuce was healed; the ulcers of the groin were granulating, and also nearly healed. Discharged the hospital at his own request.

Case LXXIX.—Thomas Connor, Nov. 1, 1814. Phymosis, profuse discharge, two small ulcers at the orifice of the prepuce. He stated that he had been disordered six months, and had not used mercury. Solut. Antim. Lot. flav. 14th, the discharge had increased, attended with pain, inflammation, and swelling of the entire penis; the lotion was discontinued, and poultices and warm fomentations directed. 22d, the pain and swelling were reduced. Lot. nig. It was found that this wash also excited pain: it was, therefore, laid aside for a solution of sulphate of zinc, in the proportion of a grain to an ounce. Under its use, and the internal exhibition of the Solut. Antim., the ulcers healed, the discharge ceased, and the patient was discharged the hospital well on the 19th December.

Case LXXX.—H. Hughes, R. H., Nov. 29, 1814. An ulcer on the external surface of the prepuce, about the size of a silver penny. He stated that he had rubbed in two ounces of ointment, which affected his mouth, but did not produce any favourable change in his complaints. Solut. Antim. Lot. flav. Dec. 20th, the ulcer had healed. Discharged the hospital.

Case LXXXI.—Charles Tully, Dec. 19, 1814. Phymosis; discharge; a circle of small ulcers round the orifice of the prepuce.

prepuce. He stated that he had been disordered three months, and had used mercury extensively, which excited a severe salivation, without producing amendment of the ulcers. Solut. Antim. Lot. flav. Feb. 13th, complaints all well. Discharged the hospital.

Case LXXXII.—John Campbell, R. H., Dec. 20, 1814. An ulcer on the external surface of the prepuce; an ulcerated bubo in each groin. He stated that he had been five weeks disordered, and had not used mercury. Solut. Antim. Sol. Sulph. Zinci pro Lot. Jan. 10th, the ulcers all healed. Discharged the hospital.

Case LXXXIII.—John Condron, R. H., April 11, 1815. An ulcer, about the size of a sixpence, situated on the external surface of the prepuce; a large bubo, containing matter, on the left groin. He stated that he had been three weeks disordered, and had not used mercury. Solut. Antim. Sol. Sulp. Zinci pro Lot. 17th, the bubo, which was opened freely by the lancet, discharged a large quantity of matter; the ulcer of the prepuce as before; the solution of sulphate of zinc was discontinued; the yellow wash directed. 23d, the ulcer of the prepuce healed; the discharge from the bubo diminished. May 8th, complaints all well; discharged the hospital.

Case LXXXIV.—Neil M'Hugh, R. H., April 14, 1815. Phymosis; several small ulcers encircling the orifice of the prepuce; discharge. He stated that he had been three months disordered; had rubbed in eighteen drachms of mercurial ointment, and taken pills, which affected his mouth, but did not produce any favourable change in his complaints. Solut. Antim. Lot. flav. 23d, the discharge was lessened, and the ulcers were healing; a bubo had appeared in the left groin, to which he was desired to rub daily half a drachm of the ointment of tartarized antimony. May 8th, ulcers healed; discharge stopped; the bubo hard, indolent, and stationary; the ointment had excited an eruption of pustules on its surface. 19th, the phymosis (which in all similar cases remains permanent after the circle of ulcers is healed) was removed by a division of the prepuce; the bubo was gradually diminishing. 23d, discharged the hospital well of his venereal complaints, but the wound of the prepuce had not healed.

Case LXXXV.—Peter Griffin, July 13, 1815. Phymosis; inflammation of the penis; discharge; three ulcers, one situated on the external surface of the prepuce, the other two on the body of the penis. He stated that he had been three months disordered, and had not used mercury. Venæsect. ad ℥xvi. Solut. Antim. Fotus. Catapl. 17th, the inflamma-

 tion tion reduced. Solut. Zinci pro Lot. 26th, the ulcers on the body of the penis healed; the other improved. August 14th, the ulcer on the prepuce also healed: its cicatrix was attended with some induration, but not that well-marked callous hardness which characterises a true chancre. Discharged the hospital, with an injunction to return in case of any recurrence of his complaints.

Case LXXXVI.—John Conroy, R. H., July 23, 1815. Three ulcers on the prepuce and glans; bubo situated over the symphisis pubis. He stated that he had been a fortnight disordered, and had not used mercury. Sol. Antim. Sol. Sulp. Zinci pro Lot. August 8th, the ulcers of the penis were healing; the bubo had ulcerated, and formed a deep cavity, with elevated edges. 20th, the ulcers on the penis had healed; that on the pubes looked foul, and its edges were still projecting: it was dressed twice a-day with lint moistened in a solution of nitrate of silver, in the proportion of two grains to an ounce. Sept. 6th, this ulcer had also healed. Discharged the hospital.

Case LXXXVII.—John Morrison, Sept. 1, 1815. Phymosis; swelling and inflammation of the penis; discharge; six ulcers on the prepuce and body of the penis: that on the prepuce, which surrounded its orifice, was the largest; the others varied from the size of a sixpence to that of a silver penny. Thirst; pulse 110; and other symptoms of high symptomatic fever. He stated that he had been two months disordered, and that he took a few mercurial pills, but not sufficient to affect his mouth. Venæsect. ad ℥xvi. Solut. Antim. Catapl. Fotus. 11th, the tension and inflammation considerably reduced; the ulcers already evinced a disposition to heal. Lot. flav. Solut. Antim. October 2d, ulcers all healed; phymosis permanent. Discharged the hospital.

Case LXXXVIII.—John Field, Sept. 1, 1815. An extensive ulcer on the body of the penis; ulcerated bubo, with elevated edges, on the right groin. He stated that he had been six weeks disordered, and had not used mercury. Solut. Antim. Sol. Sulp. Zinci pro Lot. 11th, the ulcer of the penis was healing. Oct. 2d, the ulcer on the penis and the bubo were both healed. Discharged the hospital.

I shall now proceed to the detail of the three cases which were attended with constitutional symptoms.

Case LXXXIX.—Philip Lynch, March 12, 1815. An ulcer situated on the external surface of the prepuce, the extent and size of a bean; a large ulcerated bubo, with raised edges, in the left groin. He stated that he had been six weeks disordered, and had not used mercury. Solut. Antim, Decoct. Sarsap. Sol. Zinci pro Lot. 27th, the ulcer of the penis penis was nearly healed; the edges of the bubo were, however, more raised, and projecting. April 3d, two spots, covered with thin crusts, about the size of sixpence, each raised upon a circular inflamed base, had appeared, the one on his thigh, and the other on his arm. He stated that he had only observed them three days before, at which time they resembled large pimples. Decoct. Rament. Lign. Guaic. Gum Guiac gr. v. ter. in die. The edges of the bubo were touched daily with caustic. 17th, the ulcer of the prepuce nearly healed; the edges of the bubo, which projected and overhung to a great extent, notwithstanding the daily application of caustic, were removed by the knife. 24th, a rapid amendment of the bubo had ensued since the removal of its projecting and overhanging edges; the ulcer of the penis, and the constitutional ulcers, had healed. May 22d, the bubo, which continued so long obstinate, had also, at length, cicatrized. Discharged the hospital well.

Case XC.—Henry O'Neil, June 7, 1815. The body of the penis was encircled by a series of ulcers; each about the size of a silver penny; there was also one on the external surface of the prepuce, and a large pustule, which I recognized as the commencement of one of these ulcers, on the glans penis; an incipient bubo in the right groin; a phlyzacious spot on his right side, which accurately agreed with the constitutional eruption described in the preceding case. He stated that he was five weeks disordered, that he had not used mercury, and that a fortnight before his admission the spot on his side had appeared. Solut. Antim. Sol. Sulp. Zinci pro Lot. 13th, the ulcers of the penis were healing; the phlyzacious pustule had formed a thin crust about the size of a sixpence. The bubo, which was hard and indolent, I directed to be treated by successive blisters. 26th, the ulcers of the penis were healed; the crust had fallen from the spot on his side, leaving the part healed; the bubo was almost entirely dispersed under the application of the blisters. Discharged the hospital.

Case XCI.—John Quinn, June 20, 1815. An extensive ulcer engaging the greater part of the dorsum penis, and extending up the pubes; it was covered with thick adhesive matter and attended with inflammation and swelling of the entire penis; pulse 100; thirst, severe pain, and general symptomatic fever. He stated that he was a month disordered, and had not used mercury. Venæsect ad ℥xvi. Solut. Antim. Fotus. Catapl. July 3d, the swelling and inflammation were reduced, and the ulcer which was considerably improved exhibited more decidedly the characteristic elevated edges. 11th, an eruption of pustules corresponding with the former description of phlyzacia appeared on his face and breast; he complained of pain in his head and right arm. 17th, the eruption continued to extend over his body, each spot quickly forming a crust after its appearance. The fauces were inflamed, and a superficial ulcer had appeared on the left tonsil; the ulcer on the penis was healing. Decoct. Sarsap. Vin. Antimonii gutt. xx. ter. in die. August 2d, the ulcer of the penis continued to heal, that of his throat had cicatrized; he still complained of pains in his head and arms; the spots had all formed crusts, some of which had enlarged to the extent of a shilling. The annexed drawing was taken on this day of the appearance it exhibited on his back and shoulders; in which are delineated eight phlyzacia, one at its commencement and in its progress to the formation of matter; three arrived at maturity, and terminated in apices containing matter; and four in the declining or scabbing stage. 7th, the ulcer of the penis had healed, no fresh spots of the eruption had appeared. 21st, the crusts, had fallen from the greater number of spots leaving the parts healed; he complained of pains in his head and thighs. September 1st, several fresh phlyzacia had appeared on his arms and thighs, which quickly formed crusts, covering ulcers; two of these crusts had extended to the size of half-a-crown, they were flat and of a straw colour. He was desired to discontinue the decoction and antimonial wine, and take thrice a day a pill containing three grains of antimonial powder and half a grain of calomel. 11th, the ulcers were rapidly healing, and the smaller phlyzacious pustules, after forming thin crusts on their apices, were gradually declining. 18th, the eruption had entirely disappeared, but each spot left the skin slightly discoloured. He was detained in the hospital, however, until the 30th of September. The calomel contained in the pills had not affected his mouth. The eruption in this case was more general, and the spots more numerous than I had witnessed in any other instance of this form of venereal disorder. The preceding three cases, and the two related in the appendix of my Essay, are the only instances in which I traced distinctly the ulcer with elevated edges to its constitutional eruption. But I have met with many instances of a similar eruption, in which the primary ulcers had healed before I saw the patients. The following cases may be adduced as instances of this description.

Case XCII.—Charles Gibbs, August 24, 1813. Several spots on the different parts of his body, covered with thin flat crusts like large scales, each spot or scale was about the size or rather less than a silver penny. There was but one spot which had not advanced to the scabbing state, it was a phlyzacium phlyzacium exhibiting its characteristic red inflamed and indurated base. He complained of pains in his joints, but chiefly in his shoulders, and stated that he had been disordered four years; but I could not obtain from him any satisfactory account of his complaints. Decoct. Sarsap. Solut. Antim. September 13th, the eruption had entirely declined, the crusts falling off and leaving the parts underneath healed. The pains of his joints were well; he, however, complained of pains in his side. 20th, a large phylacium had appeared on his breast; at this time, he had no other complaints. September 27th, the spots above-mentioned had formed a thin crust, and afterwards gradually declined. Discharged the hospital.

Case XCIII.—William Swift, June 20, 1814. An ulcerated bubo in the groin with projecting edges; two spots on his back, each about the size of a silver penny, and covered with a thin smooth crust; ten or twelve similar spots on his left leg and knee. He stated that five months previous to his admission, he was affected with an ulcer on the penis, which healed under a severe course of mercury, and that three weeks after the use of that medicine the eruption began to make its appearance. Decoct. Sarsap. Solut. Antim. July 4th, the crusts had fallen from many of the spots, leaving the parts healed, while several fresh phlyzacious pustules made their appearance, and quickly formed their crusts the size of the former spots. 18th, the disease continued in the same state, the older spots drying up and new ones making their appearance. The ulcer of his groin had improved. Decoct. Sarsap. Sol. Muriat. Hydrarg. August 1st, he continued in the same state. 8th, the eruption was declining, as no fresh spots had appeared for some time. 26th, discharged the hospital well.

Case XCIV.—John Duyer, April 5, 1815. Several pustules, answering the description of phlyzacia, were situated on his thighs, back, and arms, and interspersed with ulcers, covered with thin flat crusts scarcely raised above the adjoining surface. The largest of those ulcers was the size of a shilling, from which the crust had fallen off, and exposed an excavated surface without granulations. There was also a scabby eruption over the entire scalp, and an ulcerated appearance at the angle of his mouth; an ulcer of a white apthous appearance, the size of a silver penny, was situated on the back of the pharynx, and another of similar character on the right tonsil. He complained of pain in his head only, and stated that nine months previous to his admission, he was effected with an ulcer on the glans penis, which healed while he was taking mercurial pills; that six months afterwards wards the eruption first appeared on his scalp, and afterwards extended over his body. Decoct. Sarsap. Solut. Antim. 17th, the ulcers had all a granulating improved appearance, and many of the smaller ones had actually healed, the eruption was declining, and no fresh spots had appeared. 24th, the ulcers of his throat and lip had healed, and also many of those on his body. May 1st, all his complaints well. Discharged the hospital. The rapid recovery of this man from such a complication of secondary symptoms, merely from the use of Sarsaparilla and Antimonials is very remarkable.

Case XCV.—Henry Shaw, April 20, 1815. There were several spots on his body of phlyzacious character; two circular ulcers on his thigh, each about the size of half-a-crown, of a foul appearance, without granulations, raised at their edges; there were three or four round granulating ulcers on his scalp, and an ulcer of phagedenic character situated on the gum of the upper jaw, extending round the roots of three incisores, which were loose in their sockets. The fauces were inflamed; the edge of the velum and uvula was ulcerated, and there was also an ulcer of a white apthous appearance on the back of the pharynx; his mouth was severely affected by mercury at the time of his admission. He stated that he had an ulcer on the prepuce four months previous to his admission, for which he rubbed in five drachms of mercurial ointment, and that the ulcer shortly afterwards healed; that two months after the healing of the primary ulcer, the eruption began to appear, and that he had been taking mercurial pills from that period to the time of his admission. I did not order him any medicine, deferring it until the mercurial irritation should subside. May 1st, all the ulcers had improved, including that of his throat, which was very extensive. Decoct. Sarsap. Solut. Antim. 15th, the ulcers continued to improve and were healing, except that situated on his gum, which had extended; to this he was desired to apply frequently a wash, containing muriate of mercury in the proportion of gr. ſs. to ℥i. 22d, many of the ulcers had healed, that of his gum only had not improved. I directed that two of the incisores which were more loose than the others should be extracted, and that he should frequently use a lotion, containing borax and honey. The Decoction and Antimonial Solution as before. June 5th, the ulcer of his gum, which improved rapidly after the extraction of his teeth, had healed. Being in every respect well, he was discharged the hospital.

Case XCVI.—Patrick Donnelly, August 17, 1815. Numerous ulcers on his arms, thighs, and legs, varying from the size size of a silver penny to that of a shilling, and interspersed with many small spots, covered with thin scales or crusts; there were also several similar spots on the scrotum and body of the penis, which, from their appearance and the history of the case, were decidedly *secondary* and not primary ulcers. There was also a scabby eruption on the scalp. He stated that he had been affected with an ulcer on the penis two years previous to his admission, for which he underwent a severe mercurial course of three months' duration before the ulcer healed; that a few months afterwards, the eruption appeared first on his head, and afterwards on the other parts of his body, for which he submitted to another course of mercury, and continued to use that medicine almost without intermission, until the time of his admission. Mist. Acid. Nitros. Lot. flav. September 1st, the ulcers were healing. 18th, they were all healed; discharged the hospital.

The only class of those disorders resembling syphilis which remains for consideration is, that comprising the phagedenic and sloughing ulcers, and the constitutional symptoms arising from them. It was my intention to have put together such facts as I have collected with respect to these topics, and terminated the subject in this paper. But it has increased in dimension far beyond my expectations, and being obliged to postpone the sequel to another number, I shall probably avail myself of a longer period than occurs between your publications, in hopes of being able to treat of this most untractable and perplexing class of complaints in a manner more satisfactory to the public than is at present in my power. My stock of facts is increasing, and I believe (I trust not too sanguinely) that I am approximating to a more appropriate and adequate mode of treatment than the very inefficient one, at present our only resource. If I succeed, the delay will be of so much benefit to the public; if I fail, they can at any time be put in possession of all the information I can give them on the subject.

*Dublin;*

*October* 9, 1815.

---

We regret that the extreme difficulty, as well as danger, of prevailing on expert artists to hurry themselves, prevents our adding the print of a drawing which accompanied this valuable paper. As, however, we are promised a further communication from the same source, with, we hope, another drawing; the contrasting different appearances may prove the means of a more perfect comprehension in the minds of our readers.—Edit.

